# Evolutionary responses of marine organisms to urbanized seascapes

**DOI:** 10.1111/eva.13048

**Published:** 2020-07-15

**Authors:** S. Elizabeth Alter, Laraib Tariq, James Keanu Creed, Emmanuel Megafu

**Affiliations:** ^1^ Department of Biology & Chemistry California State University, Monterey Bay Chapman Academic Science Center Seaside CA USA; ^2^ Department of Biology York College City University of New York Jamaica NY USA; ^3^ Department of Ichthyology American Museum of Natural History New York NY USA

**Keywords:** adaptation, coastal development, gene flow, marine pollution, ocean sprawl

## Abstract

Many of the world's major cities are located in coastal zones, resulting in urban and industrial impacts on adjacent marine ecosystems. These pressures, which include pollutants, sewage, runoff and debris, temperature increases, hardened shorelines/structures, and light and acoustic pollution, have resulted in new evolutionary landscapes for coastal marine organisms. Marine environmental changes influenced by urbanization may create new selective regimes or may influence neutral evolution via impacts on gene flow or partitioning of genetic diversity across seascapes. While some urban selective pressures, such as hardened surfaces, are similar to those experienced by terrestrial species, others, such as oxidative stress, are specific to aquatic environments. Moreover, spatial and temporal scales of evolutionary responses may differ in the ocean due to the spatial extent of selective pressures and greater capacity for dispersal/gene flow. Here, we present a conceptual framework and synthesis of current research on evolutionary responses of marine organisms to urban pressures. We review urban impacts on genetic diversity and gene flow and examine evidence that marine species are adapting, or are predicted to adapt, to urbanization over rapid evolutionary time frames. Our findings indicate that in the majority of studies, urban stressors are correlated with reduced genetic diversity. Genetic structure is often increased in urbanized settings, but artificial structures can also act as stepping stones for some hard‐surface specialists, promoting range expansion. Most evidence for rapid adaptation to urban stressors comes from studies of heritable tolerance to pollutants in a relatively small number of species; however, the majority of marine ecotoxicology studies do not test directly for heritability. Finally, we highlight current gaps in our understanding of evolutionary processes in marine urban environments and present a framework for future research to address these gaps.

## 1 INTRODUCTION

Much of the world's population is concentrated along coasts. More than 40% of the world's population lives within a narrow coastal band, and urban density near the sea is predicted to continue to rise in future decades (McGranahan, Balk, & Anderson, 2007; Small & Nicholls, 2003). Three‐quarters of large cities and nearly all megacities (those with >20 million residents) are located on coastlines (UN Habitat, 2009) with extreme consequences for bordering marine habitats. The reach of urban influences, including artificial structures, runoff, industrial dumping, and sewage, extends beyond the terrestrial cityscape to impact coastal organisms and ecosystems. Habitat disturbance or elimination is inherent in waterfront construction including land reclamation, jetties, sea walls, and other forms of shoreline hardening. For example, it is estimated that between one‐third to one‐half of all vegetated intertidal habitats (e.g., estuaries and seagrass meadows) have been lost globally (Barbier et al., 2011; Duarte, Losada, Hendriks, Mazarrasa, & Marbà, 2013; Perkins, Ng, Dudgeon, Bonebrake, & Leung, 2015). Industries that depend on the marine environment (fishing, marine extraction, shipping) also tend to be concentrated near urban centers. These changes and inputs affect biological communities, with important consequences for processes that drive both marine ecosystem function and microevolutionary processes. However, most studies on urban evolution have focused on terrestrial systems (Alberti et al., 2017; Donihue & Lambert, 2015; Johnson & Munshi‐South, 2017; McDonnell & Hahs, 2015; Miranda, Schielzeth, Sonntag, & Partecke, 2013). In contrast, there are relatively few studies that explicitly address the microevolutionary consequences of urbanization in marine ecosystems that fringe cities.

Urbanization results in a highly altered set of selective pressures and environmental variables that may affect demography for marine taxa and vary substantially across the type of impact and the organism. In this review, we consider observed and potential evolutionary changes that are associated with urban environments. While many of the most well‐studied examples come from ecotoxicology and the strong selective pressures exacted by concentrated pollutants such as heavy metals, urban impacts such as habitat alteration can affect other aspects of microevolution, such as gene flow and genetic drift.

**FIGURE 1:**
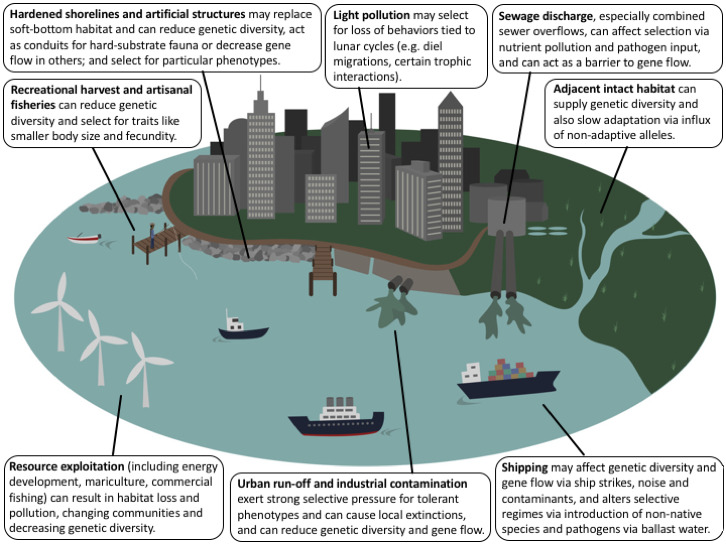
Mosaic of urban impacts on evolutionary processes including pollution, ocean sprawl, and extractive activities. These impacts can vary widely across space (from localized to widespread) and time (from short to long time frames)

As in terrestrial systems, urban impacts on marine species are extensive and varied (Figure 1). Previous studies have grouped urban drivers in marine systems into three primary categories, with potentially nonexclusive effects on microevolution: pollution, ocean sprawl, and resource exploitation (e.g., Todd et al., 2019). Pollution from point and nonpoint sources can cause contamination of sediment and water and can exert strong selective pressures on populations. Ocean sprawl encompasses the construction of artificial structures, which often represent a complete habitat replacement compared to the soft sediment that they are frequently built on, and as such can massively alter ecological communities and connectivity (e.g., Bishop et al., 2017; Firth, Browne, Knights, Hawkins, & Nash, 2016), as well as genetic diversity and structure across populations (Henry et al., 2018). Resource exploitation, which includes fisheries (both commercial and artisanal/recreational), mariculture, sediment dredging, mineral extraction, and oil and gas development, might not appear as an important urban phenomenon at first glance, but are these activities are often much more intensive near urbanizing coastal ecosystems, and especially in the case of fisheries or shellfish harvesting, represent earlier historical impacts (Kirby, 2004). Mosaics of these stressors are typically found near urbanized coastlines, resulting in heterogeneous selective regimes across relatively small spatial scales (Breitburg, Hondorp, Davias, & Diaz, 2009). In this synthesis, we maintain these categories as a framework and expand on their potential and documented effects on microevolution. We also consider how interactions between particular impacts may act synergistically to shape evolutionary processes in marine organisms.

In general, marine species span the spectrum of life‐history characteristics that affect how rapidly they are able to evolve, including generation time, reproductive mode, and effective population size (e.g., Ansell, Gibson, Barnes, & Press, 1994; Ayre, Minchinton, & Perrin, 2009; Hedgecock, 1994). Many marine invertebrates and some fishes have relatively short generation times, allowing populations to adapt more quickly to changing environmental conditions. However, effective population size is often much lower than census population size in marine species due to sweepstakes reproductive success and other factors (Beaumont, 1994; Hedrick, 2005; Kalinowski & Waples, 2002; Waples, 2016), suggesting available genetic variation may not always be as great as it might appear.

### 1.1 *Marine* versus*terrestrial evolution in urban ecosystems*

In considering a framework for urban marine evolution, it is useful to consider how evolutionary processes in city‐adjacent marine systems may mirror or differ from those in urban terrestrial systems, where such processes have been better studied. Previous studies examining evolution in urban terrestrial species indicate that many populations experience accelerated genetic drift and reduced gene flow, as well as divergent selection in life‐history traits between urban and nonurban environments (reviewed in Johnson & Munshi‐South, 2017; Rivkin et al., 2019). These patterns are driven by habitat loss and/or fragmentation, presence of impervious surfaces, pollution (including air, noise, water, and light), and higher temperatures (Rivkin et al., 2019). Some studies also suggest that despite differences between cities, urbanization itself sometimes creates convergent environments that may shape similar evolutionary trends across independent instances (Groffman et al., 2014; McKinney, 2006). While urban marine evolution shares many of the same features, patterns in marine systems may not conform to terrestrial trends entirely. Parallel impacts include habitat loss and alteration (including increases in impervious or hardened substrates); light and noise pollution; and ecological effects of invasive species. It is likely that in marine, as in terrestrial, habitats, good dispersers are less likely to lose genetic diversity due to urban impacts (Bierwagen, 2007). However, population‐level responses in urban seascapes may differ from terrestrial counterparts due to contrasts in both the nature of stressors (including duration and spatial extent) and organismal characteristics, such as higher frequency of planktonic life stages and extended propagule dispersal. Impervious or hardened substrates in marine systems may be used by organisms that colonize rocky reef habitats and may even increase dispersal via stepping‐stone effects (though these habitats are often not ecologically equivalent to natural rocky shorelines (see “Ocean Sprawl” below)). In addition, some of the stressors faced by marine populations (oxidative stress, harvesting pressure) do not have direct terrestrial urban equivalents.

The central goals of this review are to summarize the evidence for microevolutionary processes in urbanized marine ecosystems from previous studies and to identify important questions and areas for future research. We have limited our review to brackish, estuarine, and marine species, as strictly freshwater species differ in characteristics such as connectivity and dispersal and often experience a different set of anthropogenic pressures. We have also limited our analysis to metazoans, as the mechanisms by which marine prokaryotic communities evolve and adapt to urban stressors also differ substantially and have been covered in other reviews (e.g., Imran, Das, & Naik, 2019). While our primary focus is on documented evolution of marine populations via changes in allele frequencies, we also make note of studies that provide secondary lines of evidence that evolution may be occurring, such as common garden experiments. We find there is excellent potential for future research efforts to build on these studies using new genomic technologies, in order to fully explore evolutionary dynamics in urbanized seascapes.

## 2 SUMMARY OF URBAN IMPACTS AFFECTING EVOLUTION IN MARINE SPECIES

### 2.1 Ocean sprawl

Ocean sprawl encompasses artificial or engineered structures in marine or coastal environments, including shipping infrastructure (berths, moorings, shipping canals), protective structures (jetties, sea walls, breakwaters), and residential or commercial waterfront construction (bridges, canal estates, waterfront condominiums). In addition, ocean sprawl includes structures associated with marine extraction (wind farms, aquaculture) that may be located farther offshore. While there is no total estimate of the global extent of ocean sprawl, several trends indicate that most of these categories are likely to increase rather than decrease. For example, rising sea levels and the increasing frequency of coastal storms due to climate change (Hinkel et al., 2014; Nicholls & Cazenave, 2010) have prompted many coastal cities to further modify and fortify shorelines through hardened structures. The shipping industry continues to dominate global transport of goods (Ehler, Zaucha, & Gee, 2019) resulting in a growing network of ports and associated infrastructure to protect these ports. As a result, >50% of shorelines have been hardened in some parts of Asia, Europe, and America (Bacchiocchi & Airoldi, 2003; Bulleri & Chapman, 2010; Lee & Li, 2013), including some of the largest cities such as New York, Hong Kong, and Sydney (Chapman & Bulleri, 2003; Gittman et al., 2015; Lam, Huang, & Chan, 2009). Because human settlements have often preferentially been built near rivers globally (Fang et al., 2018), many urban centers are constructed near river mouths or estuaries in which sedimentary bottom habitats predominate. The transformation of the coast to hard structures often has strong effects on biological communities, especially benthic organisms (Heery et al., 2017). Artificial structures can change water flow regimes, affecting the movement of larval propagules and potentially affecting water temperature, oxygen, and light exposure (Bishop et al., 2017; Gittman et al., 2015).

Ocean sprawl can affect marine evolution via several mechanisms depending on the nature of the artificial environments created, but perhaps the best‐studied effects are on genetic diversity and connectivity. Bishop et al. (2017) review the effects of ocean sprawl on ecological connectivity between marine communities, demonstrating that artificial structures can act as both barriers to and conduits for movement of species and propagules. Because of variable dispersal potential across species, the mosaic landscapes created by ocean sprawl can fragment populations for soft‐bottom taxa, as there is increasing evidence that many benthic invertebrate may disperse at small scales (e.g., Costantini, Carlesi, & Abbiati, 2013; Darling, Kuenzi, & Reitzel, 2009). Reduced gene flow near hardened structures such as sea walls has been documented in the limpet *Patella caerula* on the coast of Italy (Fauvelot, Bertozzi, Costantini, Airoldi, & Abbiati, 2009), as well as in soft corals in the Gulf of Mexico (Atchison, Sammarco, & Brazeau, 2008; Sammarco, Brazeau, McKoin, & Strychar, 2017). However, artificial structures can also create a stepping‐stone effect for hard‐substrate fauna, creating gene flow between habitats that were previously unconnected (Coolen et al., 2020; Fowler et al., 2019). This stepping‐stone model suggests that along urbanized coastlines, species that specialize on hard substrates would be able to move between natural habitats more rapidly due to the construction of artificial hard substrate in urban coastal areas. Several studies on oil and gas platforms have shown low levels of differentiation between structures (corals; Sammarco, Brazeau, & Sinclair, 2012; polychaetes, Fauvelot, Costantini, Virgilio, & Abbiati, 2012). Coolen et al. (2020) combined particle tracking models with genetic data to show that populations of the blue mussel *Mytilus edulis* were able to use offshore oil and gas installations as stepping stones to expand their range. However, they found high genetic differentiation among some of the structures, indicating populations were colonized by few individuals (founder effects); moreover, discrepancies between modeling and genetic results suggested that patterns of differentiation were shaped both by average migration patterns and by rare events such as storms. While oil and gas platforms are not themselves structures that are typically associated with urbanization, they bear similarities to many urban structures in that they represent hardened, artificial habitat replacing soft‐bottomed habitat, and therefore, studies of these habitats may yield useful insights for predicting evolutionary patterns in urban environments.

Artificial structures also create new selective regimes for organisms whose natural habitat might be rocky reefs or shores, as they differ from these habitats in terms of surface complexity, material and rugosity, movement, and disturbance frequency and extent (Airoldi & Bulleri, 2011; Bulleri & Chapman, 2004, 2010; Chapman & Blockley, 2009; Moreira, Chapman, & Underwood, 2006; Tyrrell & Byers, 2007); moreover, as they are frequently colonized by opportunistic and non‐native species, they can introduce new predators, food sources, or parasites (Airoldi, Turon, Perkol‐Finkel, & Rius, 2015; Bracewell, Robinson, Firth, & Knights, 2013; Connell, 2001; Dafforn, Johnston, & Glasby, 2009; Firth et al., 2016; Glasby, Connell, Holloway, & Hewitt, 2007). Moreira et al. (2006) found that despite the fact that *Siphonaria denticulata* (pulmonate limpets) are abundant on sea walls in Sydney harbor, individuals are small in size and the reproductive output of the population is lower, suggesting this artificial habitat may be selecting for these features over evolutionary time scales. A study of hydroid assemblages in harbor sea walls versus natural rocky cliffs demonstrated significant differences between the two habitats, showing that harbor species were more likely to be small, short‐lived, and with a free‐swimming medusa phase—in other words, the harbor environment appears to select for r‐strategists (Megina, González‐Duarte, López‐González, & Piraino, 2013). Moreover, Turon, Tarjuelo, Duran, and Pascual (2003) showed that populations of the ascidian *Clavelina lepadiformis* living in harbors in the Mediterranean were more closely related to Atlantic coast populations than to those living outside harbors on rocky cliffs, raising the possibility that this cryptic invasion was facilitated by preadaptation to turbid and polluted conditions.

Artificial structures also change water flow, concentrating pollutants such as metals and nutrients and creating additional selective pressures. For example, swing moorings in Sydney Harbor have been shown to cause localized areas of concentrated heavy metals and differences in sediment grain size, with consequences for benthic and meiofaunal species (Hedge, Dafforn, Simpson, & Johnston, 2017). Coastal hardening can create barriers between terrestrial and marine habitats for both species who move between the two (e.g., sea turtles, terrapins, pinnipeds) and sandy beach invertebrates such as horseshoe crabs. Finally, these structures often modify wave energy and water flow regimes in localized areas, potentially creating new selective regimes in neighboring habitats (reviewed in Heery et al., 2017).

### 2.2 Pollution

Urbanization of coastal areas is nearly always associated with increased pollution in marine ecosystems. Numerous studies have demonstrated a decline in sediment pollution with distance from urban centers for many toxicants (heavy metals (Qiao, Yang, Gu, & Zhao, 2013), sediments (Todd, Ladle, Lewin‐Koh, & Chou, 2004), marine debris (Andrades, Martins, Fardim, Ferreira, & Santos, 2016; Evans et al. 1995), and PAHs (Assunção, Frena, Santos, & dos Santos Madureira, 2017). Understanding how pollutants drive microevolution in urbanized marine ecosystems is complicated by the fact that these toxicants rarely occur in isolation; most often, industrial runoff and effluents represent a complex mixture of contaminants. In older cities, sediments may harbor a wide array of chemicals that were deposited decades or centuries ago, many now restricted or banned. Evolutionary responses to these mixtures may involve many different genes and molecular pathways, possibly involving adaptive variation across many different loci. Despite these complexities, and in contrast to terrestrial urban studies, a number of studies have explored the effects of pollution on marine urban evolution. This may be because the extent of point‐source pollution in urbanized marine environments is more extensive and concentrated than it is on land and thus more likely to cause highly visible acute effects and strong selective pressures. An additional consideration is that high gene flow of marine species compared to many terrestrial taxa can lead to an influx of alleles allowing greater potential to rapidly adapt to pollutants; on the other hand, gene flow can also have a homogenizing influence, slowing evolution by introducing a steady flow of nonadaptive alleles (see below).

The potential effects of pollutants on microevolution include adaptation (evolved resistance), pollutants acting as mutagens (López‐Barea & Pueyo, 1998), reduction of genetic diversity through demographic bottlenecks and local extinction (Ma et al., 2000), and reduced gene flow due to habitat fragmentation from localized pollution events (McMillan et al., 2006; Virgilio & Abbiati, 2004a; Virgilio et al., 2003) (see Tables 1 and 2). In addition, long‐term pollution can cause large‐scale changes in biological communities from the bottom to the top of food webs. For example, contaminants of all classes were found in a meta‐analysis to reduce both richness and evenness in marine communities (Johnston & Roberts, 2009).

**TABLE 1:** Studies demonstrating changes in genetic diversity or structure associated with urban pressures in marine or estuarine species

Species	Urban stressor	Region	Method	Effect on genetic diversity	Reprod characteristics	Comments	Study
Invertebrates							
Mussel *(Mytilus galloprovincialis)*	Pollutants from industrial and boating activity	S. California	RAPD	Decrease	Planktonic larvae		Ma, Cowles, and Carter (2000)
Oyster drill *(Urosalpinx* spp)	Oil spill	mid‐Atlantic coast	allozymes	Decrease	Direct benthic development		Cole (1978)
Mussels (*Mytilus* spp)	Pollution (heavily trafficked areas)	Adriatic coast	microsatellites	Increase	Planktonic larvae	No effects on gene flow observed	Štambuk et al. (2013)
Polychaete worm (*Hediste diversicolor*)	Heavy metals (mercury)	Adriatic coast	allozymes	No difference	Limited dispersal	Several genes examined showed structure corresponding to pollution status of sites, while others showed structure unrelated to pollution	Virgilio and Abbiati (2004a)
Harpacticoid copepod (5 species)	Offshore platform	Gulf of Mexico	mtDNA	Decrease	Short generation times and direct benthic development		Street and Montagna (1996)
Limpet (*Patella caerulea*)	Artificial habitat	Adriatic coast	microsatellites	Decrease	Planktonic larvae		Fauvelot et al. (2009)
Serpulid worm (*Pomatoceros triqueter*)	Offshore platform	Adriatic coast	MtDNA	Decrease	Pelagic larvae (15 days–4 weeks)		Fauvelot et al. (2012)
Amphipod (*Talitrus saltator)*	Trace metals contamination (Hg, Cd, and Cu)	Poland (Baltic sea)	fluorescent Inter‐Simple sequence repeats (fISSRs)	Decrease	Egg broods carried by females	Populations from sites with high Hg availability had the lowest values of genetic diversity	Ungherese et al. (2010)
Giant clam (*Tridacna crocea)*	Fishing, coastal development, and sediment pollution	Singapore	mtDNA	No difference	Pelagic larvae (~1 week); long‐lived		Neo and Todd (2012)
Giant clam (*Tridacna squamosa*)	Fishing, coastal development, and sediment pollution	Singapore	mtDNA	Decrease	Pelagic larvae (~1 week); long‐lived		Neo and Todd (2012)
Mud crab (*Scylla serrata*)	Trace metals	Dar Es Salaam	mtDNA/microsatellites	no difference	Larval duration ~ 20 days	Low effective population size at the site is probably due to habitat loss and increased exploitation resulting from rapid population growth. The low effective population size at sites 7 and 8 is probably due to habitat loss resulting from extensive salt farming. The sites were located in mangrove forests affected by salt farming activities.	Rumisha, Mdegela, Gwakisa, and Kochzius (2018)
Gastropod (*Littorina brevicula)*	Heavy metals	Korea	mtDNA	decrease	Pelagic larvae (several weeks)	Differences in population haplotype diversity and structuring were found within ND6 mtDNA between polluted and unpolluted environments	Kim, Rodriguez‐Lanetty, Suh, and Song (2003)
Crab (*Pachygrapsus marmoratus)*	Heavy metals	Tuscan coast	microsatellites	decrease	Larval duration ~30 days		Fratini, Zane, Ragionieri, Vannini, and Cannicci (2008)
Barnacle (*Balanus glandula*)	Pollutants from industrial and boating activity	S. California	RAPD	decrease	Pelagic larvae (duration 3–4 weeks)		Ma et al. (2000)
Green‐lipped mussel (*Perna viridis*)	Heavy metals	Peninsular Malaysia	allozymes	increase	Larval duration ~10–21 days	Mussels from contaminated site showed the highest % of polymorphic loci	Yap, Tan, Ismail, and Omar (2004)
Variegated scallops (*Mimachlamys varia*)	Metals and organic contaminants	France	mtDNA	decrease	Pelagic larvae		Breitwieser et al. (2016)
Harpacticoid copepod (*Attheyella crassa*)	Copper and contaminated sediments	Baltic Sea	AFLP	decrease	Short generation times and direct benthic development		Gardestrom et al. (2008)
Gastropod (*Littoraria* *subvittata*)	Mangrove habitat loss due to salt farming	Tanzania	mtDNA	decrease	Pelagic larvae (several weeks)	Reduced Ne observed in most samples from mangrove sites at salt ponds compared with natural mangrove sites.	Nehemia, Huyghe, and Kochzius (2017)
Fiddler crab (*Austruca occidentalis*)	Mangrove habitat loss due to salt farming	Tanzania	mtDNA/microsatellites	decrease	Larval duration ~28 days		Nehemia and Kochzius (2017)
Prawn (*Leander intermedius*)	Lead smelter effluent	Australia/Experimental	RAPD	no difference	Unknown		Ross, Cooper, Bidwell, and Elder (2002)
Tiger prawn (*Penaeus monodon*)	Trace metal pollution	Tanzania	mtDNA	decrease	Unknown		Rumisha et al. (2017)
Isopod (*Platynympha longicaudata*)	Lead smelter effluent	Australia/Experimental	RAPD	decrease	Brood fertilized eggs		Ross et al. (2002)
Gastropod (*Littorina littorea*)	Heavy metal pollution	The Netherlands	RAPD	no difference	Pelagic larvae	Genetic structure observed across sites	De Wolf et al. (2004)
Sea star (*Patiria miniata*)	Wastewater	S. California	mtDNA/microsatellites	decrease	Pelagic larvae	Genetic structure observed across sites	Puritz and Toonen (2011)
Blue mussel (*Mytilus edulis trossulus*)	Harbors and sewage treatment plants	Baltic Sea	AFLP	no difference	Pelagic larvae	Genetic structure observed across harbors but not sewage plants	Larsson et al. (2016)
*Fishes*							
European flounder (*Platichthys flesus)*	Chemical load (pesticides)	Western Europe	gene sequencing	decrease	Spawn in water column		Marchand et al. (2010)
Atlantic killifish (*Fundulus heteroclitus*)	PCBs	US Atlantic coast	AFLPs	no difference	Benthic spawners with small home range	Strong structure between populations	Roark, Nacci, Coiro, Champlin, and Guttman (2005); McMillan, Bagley, Jackson, & Nacci, (2006)
Toothcarp (*Aphanius fasciatus)*	Urban and aquaculture water discharge	Italy	allozymes	decrease	Open water/substratum egg scatterers		Maltagliati (2002)
Toothcarp (*Aphanius fasciatus*)	Urban and aquaculture water discharge	Italy	allozymes	decrease	Open water/substratum egg scatterers		Cimmaruta, Scialanca, Luccioli, and Nascetti (2003)
European eel (*Anguilla anguilla*)	Heavy metals	Belgium	allozymes and microsatellites	decrease in allozyme diversity, no difference in msats	Catadromous with extended larval period		Maes, Raeymaekers, Pampoulie, Seynaeve, Goemans, Belpaire, and Volckaert (2005)

**TABLE 2:** Examples of rapid adaptation of marine and estuarine species to urban stressors

Species	Urban stressor	Region	Comments	Study
Invertebrates				
Ascidian (*Styela plicata*)	Copper	Australia/Experimental	Demonstrated genetic basis to resistance and GxE interactions	Galletly et al. (2007)
Bryozoan *(Watersipora subtorquata)*	Copper	Sydney	Found no difference in tolerance between polluted and reference sites, but verified significant G × E interaction	McKenzie et al. (2011)
Polychaete worm *(Limnodrilus hoffmeisteri)*	Cadmium and nickel	Hudson River	Heritable differential tolerance between populations	Klerks and Levinton (1989)
Isopod (*Platynympha longicaudata*)	Lead smelter effluent	Australia/Experimental	Heritable differential tolerance between populations	Ross et al. (2002)
Oyster (*Crassostrea gigas*)	Pesticides	Western Europe	Loci associated with resistance identified	Moraga and Tanguy (2000)
Gastropods (*Monodonta turbinata, M. turbiformis (Trochidae), Littorina punctata, L. neritoides* (Littorinidae), *Cerithium scabridum, C. rupestre* (Cerithiidae))	Inorganic (heavy metals) and organic (detergents and crude oil) pollutants	Mediterranean Sea	For all species pairs, the species with higher genetic diversity was found to be more resistant to all pollutants	Nevo et al. (1986)
Gastropod (*Littorina saxatilis*)	Heavy metals	Isle of Man, UK	Heritable differential tolerance between populations	Daka and Hawkins (2004)
Gastropod (*Crepidula convexa*, *C. fornicata*)	Copper	New Bedford Harbor, MA, USA	Hypothesizes that differences in larval transport are responsible for differential adaptation to heavy metals	Untersee and Pechenik (2007)
Mussel (*Mytilus edulis*)	Copper	Wales, UK	Heritable differential tolerance between populations	Hoare, Beaumont, and Davenport (1995)
Polychaete worm (*Hediste diversicolor*)	Copper	Adriatic Sea	Individuals with certain genotypes at loci ALD and PGI had lower mortality when exposed to copper	Virgilio and Abbiati (2004)
Copepod (*Tigriopus californicus*)	Tributyltin oxide, copper	S. California	Experimental populations exposed to contaminants showed increasing tolerance over 12 generations	Sun et al. (2014)
Fishes				
Atlantic killifish (*Fundulus heteroclitus*)	PCBs, PAHs, methylmercury, and others	Atlantic coast of US	Heritable differential tolerance between populations	Reviewed in Whitehead et al. (2017)
Gulf killifish (*Fundulus grandis*)	Oil spill	Gulf of Mexico	Rapid adaptation to pollution in Gulf killifish, apparently enabled by introduction of a non‐native congener within the last 30 generations	Oziolor et al. (2019)
Atlantic tomcod (*Microgadus tomcod*)	PCBS	Hudson River	Rapid adaptation via loss‐of‐function mutation	Wirgin et al. (2011)
Three‐spined stickleback (*Gasterosteus aculeatus*)	Pulp mill effluent	Baltic Sea	Outlier loci demonstrate selection on natural populations of G. aculeatus, causing convergence in genotypes	Lind and Grahn (2011)
Macroalgae				
Brown algae (*Fucus serratus*)	Copper)	UK	Heritable differential tolerance between populations	Nielsen et al. (2003)

Some of the best‐documented examples of marine evolution in polluted urban environments come from U.S. marine or estuarine Superfund sites such as the Hudson River and New Bedford Harbor; many focus on tolerance to organic pollutants (e.g., PCBs) and heavy metals. Toxic chemical pollutants can come from runoff, industrial dumping, as well as shipping, and include metals, tributyltin (an anti‐fouling compound), PBDEs, PCBs, PAHs, plastics, and microplastics (Cole, Lindeque, Halsband, & Galloway, 2011; Dachs & Méjanelle, 2010; Kennish, 1997; Tayeb, Chellali, Hamou, & Debbah, 2015). Many of these chemical pollutants bioaccumulate, affecting reproduction and basic cellular processes.

Sewage runoff can cause both nutrient pollution and high inputs of pathogens and parasites. In many urban settings in North America and Europe, runoff from combined sewer overflow pipes (CSOs), in which storm water and human sewage are merged and discharged into waterways, is a particularly acute issue that can result in significant ecosystem impacts. These effects include long‐term eutrophication, oxygen depletion and ammonia peaks (Borchardt & Sperling, 1997), localized acidification (Howarth et al., 2011), increases in PAHs (Launay, Dittmer, & Steinmetz, 2016), organochlorines and wastewater micropollutants (e.g., estrogens, caffeine, ibuprofen) (Ellis, 2006; Phillips et al., 2012), and exposure to human and animal pathogens (Calderón, Porter‐Morgan, Jacob, & Elkins, 2017). CSOs have been shown to impact the structure and diversity of benthic communities (Seager & Abrahams, 1990) in some cases, whereas no effects have been identified in others (Rochfort et al., 2000). Pollution from outfalls can hinder larval dispersal and thus gene flow by creating localized environments that are toxic or deadly to larvae (Puritz & Toonen, 2011) or may cause local directional selection for certain genotypes (Larsson et al., 2016). While no previous studies appear to have examined adaptation of metazoan communities near CSOs, these sources of contaminants and nutrients are likely to select for organisms that are able to tolerate a wide range of chemical stressors. Benthic, stationary species such as mollusks are most likely to be affected by CSOs as they will experience the most direct effects; however, mobile species may also be impacted by increases in pathogens, especially where the zone of CSO impacts overlaps with feeding and breeding grounds. For example, sea otters and pinnipeds have been shown to be susceptible to parasites and diseases from urban runoff (Miller et al., 2010; Stoddard et al., 2008).

Beyond industrial or chemical toxicants, urban pollution of marine ecosystems extends to many other sources that are less well‐studied. Very few studies, if any, have addressed the effects of these nonchemical forms of pollution on urban marine evolution, though they may often exert strong selective effects. Sediment pollution (an increase in sediment in the water column) can result from terrestrial activities such as deforestation, or marine activities such as dredging and construction, and results in increased turbidity and smothering of benthic organisms (Junjie, Browne, Erftemeijer, & Todd, 2014; Rogers, 1990). Shoreline industrial operations (e.g., cooling plants) can also create localized brine and thermal pollution. Additional aspects of pollution that are increasingly common, if less‐studied, include noise pollution and light pollution. Noise pollution can affect survival and reproductive processes for animals that depend on sound for finding mates and food (e.g., cetaceans, oyster toadfish) and can negatively impact species that are sound‐sensitive (bivalves: Charifi, Sow, Ciret, Benomar, & Massabuau, 2017; Mosher, 1972; Peng et al., 2016; Roberts, Cheesman, Breithaupt, & Elliott, 2015), cephalopods (Fewtrell & McCauley, 2012), and shrimp and other invertebrates (Solan et al., 2016). Light pollution is another widespread phenomenon associated with coastlines; a recent study estimated 22.2% of the world's coastlines (excluding Antarctica) were exposed to regular artificial light pollution in 2010 (Davies, Duffy, Bennie, & Gaston, 2014). Artificial light sources from urban centers and harbors can mask natural lunar cycles that marine species have evolved with and have been shown to affect important processes such as diel migrations (Moore, Pierce, Walsh, Kvalvik, & Lim, 2000), navigation (Merkel & Johansen, 2011; Tuxbury & Salmon, 2005), trophic interactions (Becker, Whitfield, Cowley, Järnegren, & Næsje, 2013; Bolton et al., 2017; Underwood, Davies, & Queirós, 2017), photosynthesis (Ayalon, de Barros Marangoni, Benichou, Avisar, & Levy, 2019), and communication (Davies et al., 2014; Gaston, Bennie, Davies, & Hopkins, 2013). No studies have examined evolutionary consequences of light pollution but we speculate it would select for alleles that unlink these critical life processes from lunar cycles (or other light cycles) and could affect recruitment and gene flow between populations by changing migration patterns that evolved under predictable lunar and stellar regimes. For example, coastal lighting has been shown to act as an “evolutionary trap” in some taxa (Robertson & Blumstein, 2019), disorientating hatchling sea turtles and often preventing them from reaching the sea, which can result in a reduction in the number of turtle nests in areas with extensive light pollution (Mazor et al., 2013; Tuxbury & Salmon, 2005). As a result, artificial light has been implicated in reduction in recruitment in some sea turtle populations (Dimitriadis, Fournari‐Konstantinidou, Sourbès, Koutsoubas, & Mazaris, 2018). Another recent study on larval coral reef fishes showed that artificial light affected selection of settlement habitats, susceptibility to predation, growth rate, and mortality (O'Connor et al., 2019).

Evolved resistance to pollutants is the most commonly documented mode of evolution and has been studied extensively in several marine species (Table 2), with most studies focused on effects of heavy metals and, to a lesser extent, organic pollutants. A full review of evolved resistance to pollutants by marine animals is beyond the scope of this article but is addressed in many previous reviews (e.g., Amiard‐Triquet, 2019; Belfiore & Anderson, 2001; Hamilton et al., 2016; Klerks & Weis, 1987; Medina, Correa, & Barata, 2007). A number of studies have also demonstrated that evolved resistance to pollutants often comes with a fitness trade‐off (e.g., Hoffmann & Parsons, 1991, Jayasundara et al., 2017). Some of the most thorough studies of evolved resistance to pollutants have focused on the Atlantic killfish, *Fundulus heteroclitus*. Extensive physiological, genomic, and transcriptomic studies of this species have revealed specific alleles at loci involved in resistance, as well as widespread signatures of selective sweeps indicative of adaptive evolution (Reid et al., 2016; Whitehead, Clark, Reid, Hahn, & Nacci, 2017) (see Case Study below). Additional studies in fish have revealed genomic mechanisms underlying tolerance to pollutants in Atlantic tomcod *Microgadus tomcod* (Wirgin et al., 2011) and American and European eels *Anguilla* spp (Laporte et al., 2016). A number of studies have also addressed adaptation to pollutants in invertebrates. For example, a population of the benthic polychaete *Limnodrilus hoffmeisteri* evolved resistance to cadmium and nickel at a highly polluted estuary site in the Hudson River within 3–4 generations (Klerks & Levinton, 1989). Follow‐up studies then documented the loss of resistance following the cleanup of the site (Levinton et al., 2003). Numerous studies have focused on tolerance to copper (Galletly, Blows, & Marshall, 2007; Hoare et al., 1995; McKenzie, Brooks, & Johnston, 2011; Piola & Johnston, 2006a; Sun et al., 2014; Untersee & Pechenik, 2007; Virgilio & Abbiati, 2004, ), as it is particularly toxic to marine invertebrates (see Case Study below) and is often used as an anti‐foulant for that reason. A large number of studies (e.g., Daka & Hawkins, 2004; Grant, Hateley, & Jones, 1989) document differential tolerance in populations between contaminated and clean sites, but do not test whether tolerance is inherited across generations.

### 2.3 Resource extraction

While resource extraction—including fisheries and aquaculture—is perhaps less obvious as a driver of evolution in urbanized marine environments compared with ocean sprawl and pollutants, extractive activities nearly always predate the other two categories in the history of any urban settlement and generally represent the oldest anthropogenic stressors on marine systems resulting from high human density along coastlines (Jackson et al., 2001). In many cases, past exploitation of marine populations near human settlements reshaped entire ecosystems early in their histories; for example, overharvesting of fisheries on the east coast of the United States began near urban centers such as Boston and sequentially depleted neighboring areas (Kirby, 2004). Likewise, the demise of oyster reefs along urbanized coastlines in Europe, Australia, and the United States resulted from overharvesting, often combined with water pollution (Airoldi & Beck, 2007; Alleway & Connell, 2015). While commercial fishing has declined in urban areas in developed countries due to pollution and the historical legacy of overexploitation, subsistence fishing in urban coastal communities continues to be important for food security in developing countries (Garcia & Rosenberg, 2010; Malakar, Mishra, & Patwardhan, 2018). In developed regions such as the United States, Europe, Australia, recreational fishing typically exceeds commercial fishing in urban areas (McPhee, 2017). Post et al. (2002) showed that recreational angling effort radiating from urban centers is associated with declining catch rates and stock depletion. Recreational harvesting can also impact invertebrate populations on rocky shores (Airoldi, Bacchiocchi, Cagliola, Bulleri, & Abbiati, 2005). In addition to these trends in fisheries, urbanization corresponds with growing extractive industries including shoreline or nearshore dredging for sand, minerals, rock, and coral for construction or other purposes (Charlier & Charlier, 1992). Exploitation of marine populations can have well‐studied effects on the evolution of populations, including reduced effective population size, selection for smaller body sizes, earlier sexual maturity, and other life‐history traits (e.g., Conover & Munch, 2002; Hauser, Adcock, Smith, Ramírez, & Carvalho, 2002; Heino, Díaz Pauli, & Dieckmann, 2015; Kuparinen & Merilä, 2007; Therkildsen et al., 2019; Uusi‐Heikkilä et al., 2017). While a full treatment of this topic is outside the scope of this review, readers are referred to the studies cited above as well as several recent reviews (Kuparinen & Festa‐Bianchet, 2017; Palkovacs, Moritsch, Contolini, & Pelletier, 2018; Tillotson & Quinn, 2018).

Little research exists on modern urban marine fisheries in general, and few if any studies have explicitly examined evolution due to exploitation in urban fisheries. However, several studies have shown that urban fisheries can result in overexploitation and population depletion due to the high concentration of potential fishers/harvesters, especially considering that urban marine populations may experience fluctuations in population size due to environmental stochasticity (Coleman, Figueira, Ueland, & Crowder, 2004; Hartill, Cryer, & Morrison, 2005; Kraeuter et al., 2008; O’Toole, Hanson, & Cooke, 2009; Post et al., 2002). This suggests that these activities do have the potential to affect microevolution via reductions in genetic diversity and fisheries‐induced selection (Lewin, Arlinghaus, & Mehner, 2006). For example, in fishes with genetically determined seasonal migration, as in salmon, selective angling in certain seasons may interfere with adaptive structuring of populations (Consuegra, Leániz, Serdio, & Verspoor, 2005).

In addition to these three categories, urbanization near coastlines can have other impacts on evolutionary dynamics that result directly or indirectly from dense human habitation near ocean ecosystems, summarized briefly here. Disruption to salinity and sediment profiles in estuaries via canalization, withdrawal of freshwater sources, and mosquito ditching can have a long‐term impact on estuarine organisms by reducing or eliminating nursery functions or other critical ecosystem services (Courrat et al., 2009), potentially resulting in a reduction in genetic diversity, and reduced gene flow at a large spatial scale due to loss of pristine estuarine habitat along coastlines. Moreover, the density of vessel traffic near port cities can result in a host of negative ecological effects with potential to influence evolution of marine organisms. These include transport of invasive species via ballast water and other mechanisms, as well as mortality to marine megafauna (marine mammals, pinnipeds, and turtles) from ship strikes. While it is difficult to quantify the evolutionary impact of shipping, particularly for long‐lived organisms like marine mammals, ship strikes near cities are likely to have had an outsized impact on genetic diversity particularly in small, endangered populations. For example, habitat fragmentation due to increased shipping along the urbanized eastern U.S. coastline has resulted in high ship strike mortality in the North Atlantic right whale population, which numbers around 400 individuals (Mullen, Peterson, & Todd, 2013). This species has been called “the urban whale” due to the overlap in its habitat with human activities (Kraus, Rolland, & Rolland, 2007). While the long generation time of this species likely precludes genetic adaptation to urbanization, erosion of genetic diversity due to mortality from ship strikes and entanglement near cities may constrain future adaptation to changing global conditions. Finally, in some urban areas, food provisioning by humans may be changing parasite load, gut microbiome, and timing of reproductive behaviors, with implications for long‐term adaptation, as has been shown in wading birds in southern Florida wetlands (Kidd‐Weaver et al., 2020; Murray et al., 2020).

### 2.4 Interactions between stressors

The effects of anthropogenic stressors on microevolutionary dynamics cannot be considered in isolation as they may act cumulatively—additively, synergistically, or antagonistically—on marine populations (Crain, Kroeker, & Halpern, 2008; Todd et al., 2019). Results from previous meta‐analyses suggest that multiple stressors often act synergistically in marine systems, in contrast to terrestrial and freshwater ecosystems (Crain et al., 2008; Harvey, Gwynn‐Jones, & Moore, 2013). Different stressors may overlap in time, or may occur sequentially as cities grow and develop. Many stressors, particularly those that result in large‐scale habitat loss such as ocean sprawl, are likely to reduce population size substantially, which can erode standing genetic variation and hinder the ability of populations to rapidly adapt to acute stressors such as pollution. Our synthesis indicates that a wide variety of urban stressors from artificial structures to pollutants are associated with declines in genetic diversity in diverse marine species (Table 1); reduced genetic diversity may compromise the ability of these populations to adapt to other stressors. In addition to loss of genetic diversity, populations living on artificial structures may be smaller‐bodied than populations living on natural hard surfaces (Megina et al., 2013; Moreira et al., 2006). A number of studies show that smaller‐bodied individuals are more susceptible to toxic effects from pollutants (e.g., McKenzie et al., 2011), indicating that smaller‐bodied populations may be at greater risk from subsequent pollution events. Likewise, resource extraction can also interact with pollution in similar ways: Overharvesting can affect both overall abundance and genetic diversity, as well as select for reduced mean body size. Habitats compromised by pollutants can be more susceptible to invasions (Piola & Johnston, 2008).

As an example of antagonistic interactions, nutrient enrichment can lessen the harmful effects of toxins in some cases (Breitburg et al., 1998). On the other hand, prevalence of infection by parasites was found to increase in oysters that were exposed to other stressors like higher sediment load or decreased water flow (Lenihan, Micheli, Shelton, & Peterson, 1999).

Perhaps the most important cumulative effects on evolution in urban seascapes, and yet among the least studied to date, will come from the physiological challenges to marine organisms presented by climate change and ocean acidification, when combined with ongoing urban stressors. Clear examples of these complex problems come from the synergistic effects of nutrient enrichment and other stressors such as sea level rise and predator loss on estuarine salt grasses (Deegan et al., 2007, 2012). Warming waters can also lead to invasion of parasites or competitors (Stachowicz et al., 2002). Generalized stress from climate change impacts to water temperature, pH, and salinity may also reduce the ability to resist and recover from exposure to pollutants (Moe et al., 2013).

Stressors that occur at the same time can present challenges to individuals along multiple physiological axes, and timing of stressors can affect physiological and potentially evolutionary outcomes (Bible et al., 2017; Molinos & Donohue, 2010). Stressors that act in concert (simultaneously) can potentially delay or change evolutionary dynamics; for example, a study that examined the evolutionary response of bacteria to both predators and antibiotic exposure showed that the evolutionary responses to both stressors were delayed (Hiltunen et al., 2018). However, consecutive stressors can, in some cases, have larger impacts on individuals and populations than simultaneous stressors, as in the case of acidified conditions and salinity in crab larvae (Miller et al., 2014) and consecutive warmer air and water temperatures in sea stars (Pincebourde, Sanford, Casas, & Helmuth, 2012), if exposure to the first stressor causes physiological changes that leave an organism vulnerable to a second stressor (e.g., Todgham & Stillman, 2013). Jayasundara et al. (2017) showed that killifish adapted to polluted waterways demonstrated altered metabolic and physiological responses to other stressors such as temperature, indicating that adaptation to pollution may compromise the ability of this population to acclimate to other stressors. In addition, each stressor may be inconsistent and may fluctuate over time. Because no single phenotype may be optimal in the context of these multiple, fluctuating stressors, such combinations may select for broad organismal plasticity over time (e.g., Gomez‐Mestre & Jovani, 2013; Hallsson & Björklund, 2012).

## 3 CASE STUDIES: RAPID ADAPTATION IN URBAN MARINE ECOSYSTEMS

For most marine species, the speed of urban environmental change will outpace their capacity to evolve: Rapid adaptation to urban conditions is likely to be the exception rather than the rule. However, certain marine species appear to survive and even thrive in urbanized seascapes, perhaps the marine equivalents of terrestrial synanthropic species such as pigeons and raccoons. For example, the photosynthetic capacity of the green algae *Ulva lactuca* increases in environments with greater pollution, while that of the brown algae *Sargassum stenophyllum* decreases (Scherner, Barufi, & Horta, 2012). Overall, many of the species that appear to thrive in urbanized seascapes appear to have a generalized tolerance of environmental variability, which may derive in part from preurban adaptation to such variability in native estuaries: Species that are able to persist in estuarine and other tidal environments must necessarily cope with large fluctuations in variables like temperature, oxygen, and sediment load and thus show cross‐tolerance across stressors (e.g., Ravaschiere et al., 2017; Schulte, 2007; Todgham, Schulte, & Iwama, 2005). The evolution of physiological plasticity is key to allowing species to persist in challenging environments (e.g., Brennan, Galvez, & Whitehead, 2015), and thus, these species may in some cases be preadapted to stressful and variable urban conditions. In addition, adequate genetic diversity is necessary for evolutionary responses to a wide range of urban stressors. A number of studies of rapid evolution to urban stressors indicate organisms are able to adapt quickly due to standing genetic variation (see Reid et al. 2017; Wirgin et al., 2011 below), or even more intriguingly, via introgression from other species (Oziolor et al., 2019). Additional experimental evidence indicates the importance of standing variation in adaptation; for example, Nevo, Noy, Lavie, Beiles, and Muchtar (1986) showed that in several pairs of species with differing levels of genetic diversity, the species with greater diversity was found to be more resistant to toxicants. The addition of genomic data from a wider array of species may reveal more about the most common sources of genetic variation during episodes of rapid adaptation.

### 3.1 Case study: Copper tolerance in macroalgae and invertebrates

Copper is one of the most common pollutants found in industrial sediments and is among the most toxic metals for both macroalgae and invertebrates, as it can inhibit photosynthesis and disrupt important cellular functions (Connan & Stengel, 2011; Gledhill, Nimmo, Hill, & Brown, 1997; Qian et al., 2009). For this reason, it is often introduced purposefully into the marine environment as an anti‐foulant. Several species of macroalgae show population and species‐level differences in tolerance to copper. For example, Han, Kang, Park, Lee, and Brown (2008) showed that growth and photosynthetic rate were largely unchanged in response to copper exposure in one species of Ulva (*U. armoricana*), whereas both parameters decreased significantly in another species (*U. pertusa*). Nielsen, Brownlee, Coelho, and Brown (2003) found that populations of the brown alga *Fucus serratus* from copper‐contaminated sites were more resistant to copper exposure, as measured by tissue concentration and growth rates, suggesting an exclusion mechanism. Moreover, similar responses in progeny of the adults indicate that the resistant phenotype is at least partially inherited. In contrast, Brown, Newman, and Han (2012) tested for interpopulation differences in copper tolerances in the brown seaweed *Gracilariopsis longissimi* and found no significant differences between populations from habitats differing in copper concentration, suggesting that observed copper tolerance is a species‐wide feature rather than an evolutionary response. Polyploidy and the ability to hybridize may be important means by which some species of algae maintain the genetic variability needed to cope with new habitats (Coyer et al., 2006). While relatively few studies have tested genetically based and heritable tolerance in macroalgae, the variability in tolerance across populations and species indicates this system has excellent potential for future studies.

Copper is also especially toxic to many marine invertebrates such as cnidarians, mollusks, bryozoans, arthropods, and poriferans (Johnston & Keough, 2000; Reichelt‐Brushett & Harrison, 2000). In the bryozoan *Watersipora subtorquata*, heritable differences in tolerance to copper were found across individual colonies, suggesting this species has the potential to evolve rapidly (McKenzie et al., 2011). Larval size, which has a heritable component but also results from the interaction between parental genotypes and environmental conditions, was also found to positively affect tolerance in this species. Larval size has also been shown to increase in response to pollutant levels in another bryozoan, *Buguta neritia* (Marshall, 2008), indicating this trait may be a plastic response to fluctuating levels of pollutants. Galletly et al. (2007) used a quantitative genetic breeding design to examine the genetic basis of copper tolerance in the ascidian *Styela plicata* and found complex GxE interactions suggesting genetic mechanisms for tolerance differ across different exposure levels. Numerous other studies have shown differential tolerance across populations with different contaminant histories (e.g., Piola & Johnston, 2006a; Sáez et al., 2015), or across different species (Piola & Johnston, 2006b, 2009) but have not explicitly tested for heritability of these traits.

### 3.2 Case study: Atlantic killifishes (*Fundulus heteroclitus*)

The Atlantic killifish *Fundulus heteroclitus* is perhaps the best‐studied example of rapid and repeated adaptation to pollutants. Populations of this species, which is highly abundant along the eastern seaboard of the United States and Canada, have independently evolved heritable tolerance to a variety of pollutants and toxicants, including heavy metals, PAHs, and PCBs (Nacci et al., 1999; Nacci, Champlin, & Jayaraman, 2010; Reid et al., 2017; Weis, 2002; Whitehead et al., 2017,). Extensive genomic and transcriptomic data for this species reveal a complex set of genes involved in tolerance pathways, including but not limited to the aryl hydrocarbon receptor‐based signaling pathway (Reid et al., 2016). For example, deletions in AHR are observed in tolerant populations of *F. heteroclitus*, mirroring the findings of Wirgin et al., 2011 in Atlantic tomcod (*Microgadus tomcod*) from the Hudson River. The very large effective population size of this species promotes relatively high standing genetic variation, and genomewide data suggest this standing variation is likely the source for adaptive alleles. Pollution tolerance was also observed in the congener of *F. heteroclitus*, the Gulf killifish *F. grandis*, with evidence that introgressed alleles from *F. heteroclitus* may have played a role in the evolution of tolerance to PAHS, PCBs and (Oziolor, Bigorgne, Aguilar, Usenko, & Matson, 2014; Oziolor et al., 2019). These studies add to a growing body of work suggesting that interspecies introgression can be a powerful force aiding rapid adaptation to new environments.

## 4 SPATIAL SCALES OF EVOLUTION: CONSIDERATIONS FOR URBANIZED SEASCAPES

As is the case in terrestrial urban ecosystems, the temporal and spatial scale of stressors has important implications for evolutionary pressures in urbanized seascapes. Because local adaptation can only occur when the speed of selection exceeds the homogenizing influence of the inflow of alleles from nearby populations, both the scale and magnitude of selective forces, as well as the scale of gene flow, influence how quickly marine organisms are able to adapt to particular urban stressors.

Many urban impacts on marine organisms are highly localized—for example, anti‐foulant‐coated surfaces result in a very fine‐scaled mosaic of selective regimes for encrusting organisms separated by only meters (e.g., McKenzie et al., 2011), and heavy metals and other pollutants can be sequestered in sediment patches of varying sizes and exposure can vary with season due to interactions between pollutants and organic matter (Birch, Taylor, & Matthai, 2001; Buggy & Tobin, 2008). In contrast with this localized scale of stressors, many species of nearshore invertebrates and fishes have a pelagic larval stage that ranges from days to months, and a sedentary benthic adult stage, suggesting a much wider spatial scale of dispersal. This mismatch between the small scale of the selective pressure and the large potential range of gene flow suggests that adaptive processes may often be slowed in urban coastal species; indeed, there is evidence for this idea from some comparative studies across similar species with different larval dispersal (Untersee & Pechenik, 2007). However, there are many exceptions to this general model: For example, many stressors have wider‐ranging impacts within and beyond the urban coastline (shipping noise, coastal construction), and a number of marine species including some fishes, gastropods, and crustaceans have direct development or very short larval duration rather than extended larval dispersal, generally with expected consequences for genetic structure (e.g., Collin, 2001; Dawson, 2001; Kyle & Boulding, 2000). Moreover, both natural (oceanographic) and human‐made barriers to dispersal may result in gene flow occurring across smaller scales than larval duration would suggest (Marko 2004; Marko, Rogers‐Bennett, & Dennis, 2007; Pelc, Warner, & Gaines, 2009). For species that produce larvae in patchy or enclosed habitat (e.g., estuaries or lagoons), gene flow may be further reduced below expected levels (Watts & Johnson, 2004). Indeed, studies in Table 1 indicate that urbanization is often correlated with decreases in genetic diversity across a wide variety of species with different reproductive modes, suggesting that gene flow between urban and reference sites is not extensive enough to homogenize alleles.

To further complicate matters, coastal cities may themselves also generate new opportunities for dispersal between and out of urban marine ecosystems, both via creation of artificial stepping stones for hard surface dwelling organisms (see Ocean Sprawl above) and also by generating new opportunities for transport via ballast water and rafting on marine debris or tar balls. Sessile calcareous organisms such as serpulid polychaetes, bryozoans, barnacles, and gastropods are often found encrusted on floating plastic debris, and this mode of transportation has allowed, in some cases, long‐range dispersal away from native habitat (e.g., Winston, 2012) and promotes invasion by nonindigenous species (Campbell et al., 2017).

While gene flow from nearby unaffected ecosystems can slow the process of adaptation by homogenizing populations, in cases where survivorship in an urban‐impacted system is low, those source populations may harbor crucial genetic diversity that allows adaptation to occur. Thus, the proximity of urbanized seascapes to nearby intact ecosystems should be considered when studying the speed of evolution and in planning protected areas.

## 5 CONCLUSIONS, OUTLOOK, AND RECOMMENDATIONS FOR FUTURE STUDIES

As human density continues to rise near coastlines, activities ranging from ocean sprawl to pollution will exert increasing selective pressures on nearshore organisms living in urbanized seascapes. In many urban marine ecosystems, selective regimes may change quickly over time, and/or may be heterogeneous over small spatial scales, to the extent that the most evolutionarily successful species will be those with large standing genetic variation and those with high phenotypic plasticity.

It is clear that there is enormous potential for improving our understanding of how evolution is occurring in these habitats—very few studies to date have explicitly examined evolution in urban marine habitats and, those that have, have studied the effects of particular urban stressors rather than the synergistic effects of urbanization as a whole. The majority of studies that examine urban impacts on marine species test for tolerance to pollutants between urban and reference sites, but few address the heritability of traits that promote tolerance, and fewer still delve into the genetic mechanisms underlying those traits (but see exceptions such as Whitehead et al., 2017). However, the field of evolutionary studies of urban marine organisms is now poised to make rapid advances toward documenting and deciphering the mechanisms of microevolution in these ecosystems. New genomic technologies can be used to build on decades of ecotoxicological studies across various marine species, leveraging modern methods to assess selection in natural populations. For example, genome scans now permit researchers to look for selective sweeps at the population level (Nielsen et al., 2005). This method, which has been used in a variety of terrestrial species (including some urban examples, e.g., Harris & Munshi‐South, 2017) as well as marine species (Dalongeville, Benestan, Mouillot, Lobreaux, & Manel, 2018; Liu et al., 2017), can be used to compare signatures of selection between populations of urbanized and reference sites. An advantage of this strategy is that it allows a holistic examination of evolution across the suite of potential stressors in an urban environment. This approach has been taken in a small number of urban marine species previously (mummichog: Whitehead et al., 2017, Osterberg, Cammen, Schultz, Clark, & Di Giulio, 2018; oysters: Gutierrez et al., 2018, Bernatchez et al., 2019), but there is excellent potential to apply it more widely to a greater number of taxa and urban systems.

A number of important evolutionary questions remain to be answered in urban marine species. Among these are as follows: (a) Do urban pollutants appreciably increase mutation rate in marine species? Previous studies have shown an increase in mutations upon exposure to mutagens such as PCBs and PAHs (López‐Barea & Pueyo, 1998; Ohe, Watanabe, & Wakabayashi, 2004) but it is not known whether these effects are widespread enough to affect evolutionary dynamics. (b) Do marine species show convergent traits across different urban areas, similar to what has been observed in terrestrial systems? Studies of urban terrestrial environments show that species often independently converge on particular traits across different cities. Are similar “urban syndromes” evident in marine species? (c) How does spatial heterogeneity across urbanized seascapes affect the potential for organisms to evolve? (d) How does standing genetic variation and/or gene flow from nearby intact ecosystems affect evolvability of urban marine species? As noted above, a number of case studies of rapid adaptation to urbanized seascapes have suggested that adaptive alleles derived from standing genetic variation, or in some cases, from introgression from related species.

To address these questions and others, comprehensive research programs are needed to better understand evolution in urbanized seascapes across a diversity of taxa and geographic areas, including long‐range dispersers and those with limited dispersal capabilities. Such studies could combine genome scans of natural populations to detect signatures of selection, comparing populations from urbanized and nearby nonurbanized sites, with common garden and reciprocal transplant experiments to determine the evolutionary basis of fitness in the presence of urban stressors. Many of the previous studies on the effects of urban stressors on neutral dynamics (genetic diversity and structure) were carried out using very few or anonymous markers (Table 1). Newer methods such as RADseq allow the rapid assessment of genetic diversity and structure at a genomewide scale, permitting a much more thorough analysis than was previously possible and opening the possibility of more fine‐scaled demographic analyses of recent bottlenecks; however, it is important to note that RADseq methods require sufficient marker density across the genome in order to have utility in identifying adaptive regions (e.g., Catchen et al., 2017; Lowry et al., 2017a; Lowry et al., 2017b). For the purposes of studying adaptation, methods such as low‐coverage whole‐genome sequencing, Pool‐seq, exome capture, and RNAseq are superior to RADseq and could be used to expand on experimental studies such as Nevo et al. (1986) to compare tolerance and adaptive responses of populations or species with differing standing genetic variation (Hoban et al., 2016; Jones, & Good, 2016; Puritz & Lotterhos, 2018; Schlötterer, Tobler, Kofler, & Nolte, 2014).

Another valuable direction for future research would be use of historic specimens (e.g., archived scales) to analyze genomic changes over many generations and to assess hypotheses about the origin of adaptive alleles. Here again, new methodologies such as Hy‐RAD and targeted capture have improved the chances of sequencing DNA from old specimens. Of particular interest would be urban marine populations near older cities (>400 years), where evolution has had more time to occur, such as Lagos, Osaka, Jakarta, Panama City, Singapore, New York, Boston, and Sydney. However, a challenge with older cities is that, due to greater urban sprawl, it is often more difficult to find nearby and relevant reference (nonurbanized) sites for comparison.

With sea level rise, higher temperatures and increases in extreme weather events, urban dwellers, and policymakers are increasingly aware of the importance of ensuring the resilience of coastal marine ecosystems to protect coastlines and restore biodiversity (Dafforn et al., 2015; Moschella et al., 2005; Munsch, Cordell, & Toft, 2017; Perkins et al., 2015; Strain et al., 2018). However, evolutionary potential is rarely included as an explicit factor in planning marine habitat restoration and interventions. To allow thriving marine communities on the edge of dense cities, it is critical that urban planners and designers consider evolutionary processes. Shoreline development can be mandated to include habitat improvements that will help stabilize marine population sizes and therefore help maintain population genetic diversity—for example, in a meta‐analysis of 109 studies, Strain et al. (2018) found that the majority of eco‐engineering interventions in urban marine ecosystems increased the abundance of particular species and/or diversity of species. Promising directions include a new residential development along New York City's East River that includes restored oyster reefs, salt grass, and small tidal inlets. Such “blue spaces” also present great opportunities for education and community engagement to help city dwellers learn about and eventually advocate for protecting urban marine habitats (Dafforn et al., 2015). In addition to encouraging habitat improvements as part of construction projects, any development of artificial structures near cities should include consideration of how these structures may hinder or facilitate gene flow. As marine urbanization increases over the coming decades, improving our knowledge of how marine species are evolving in these altered habitats, as well as working to conserve them, will be critical to minimizing the long‐term impacts of urban pressures on bordering seascapes.

## CONFLICT OF INTEREST

None declared.
